# Drivers of conservation and utilization of pineapple genetic resources in Benin

**DOI:** 10.1186/2193-1801-3-273

**Published:** 2014-05-31

**Authors:** Enoch G Achigan-Dako, Charlotte Abike Adjé, Sognigbé N’Danikou, Nicodème V Fassinou Hotegni, Clément Agbangla, Adam Ahanchédé

**Affiliations:** Horticulture and Genetics Unit, Faculty of Agronomic Sciences (FSA), University of Abomey-Calavi, 01 BP 526 Tri postal, Cotonou, Republic of Benin; Laboratory of Genetics and Biotechnology, Faculty of Science and Technology (FAST), University of Abomey-Calavi, 01 BP 526 Tri postal, Cotonou, Republic of Benin

**Keywords:** *Ananas comosus*, Cultivation practices, Farmer’s knowledge, Genetic resources, Pineapple

## Abstract

Valuation of farmer knowledge has been seen as a route to promote sustainable use of plant genetic resources. In pineapple production systems in Benin, inadequate knowledge of cultivation practices can lead to a number of inconveniences including abandon of some varieties and cultivars. To understand how farmer’s knowledge and cultivation practices impact the sustainable utilization of pineapple genetic resources, we surveyed 177 pineapple farmers in southern Benin. We assessed farmers’ knowledge and analyzed the relationship between their knowledge and factors such as age, education, and locality of provenance. Pineapple production system was dominated by men (96% respondents). According to farmers, Smooth cayenne is international market-oriented while Sugarloaf mainly targets domestic and regional markets. All farmers recognized that Smooth cayenne provided more income (USD 5,750/ha) than sugarloaf (USD 3,950/ha) in the production systems of southern Benin. The high value of median scores in comparison with the range of possible score showed that most farmers agreed and shared relatively similar knowledge. Correlation matrix and multiple linear regressions showed a significant relationship between farmers’ practices and their knowledge of the plant; their knowledge of pineapple varieties is based on fruits traits. Also, farmer’s knowledge was associated with locality of provenance. Constraints and options for genetic resources conservation and utilization in the pineapple production systems in Southern Benin were discussed based on current knowledge.

## Introduction

Crop genetic diversity serves to buffer environmental constraints and to sustain traditional farming systems (Gepts 2006; Samberg et al. 2013). However, in intensive production systems sustainable utilization of genetic diversity has frequently been at risk (Clement 1999) when clear conservation strategies (e.g. seed genebanks, field genebanks, on-farm conservation, reserves) are not available. The search for homogenous and high yielding varieties, with their associated bulk of agricultural inputs (e.g. chemicals, farm machinery, irrigation) and the development of markets are still threatening the reliance of farmers upon genetic diversity (Swanson and Goeschl 2000) when elite cultivars are promoted. This debate started some years back and effort has been put to promote the ‘*conservation through use*’ approach (Jarvis et al. 2000). Such approach is appropriate when decision makers and researchers have adequate knowledge of the genetic resources available and farmer’s criteria for variety selection which are key to promoting effective plant breeding (Temudo 2011) and on-farm conservation. However, a major driver of the ‘*conservation through use’* approach is the value (e.g. social, nutritional, economical, nutraceutical, ecological) farmers assign to crop genetic resources (Brush and Meng 1998), be they modern or traditional varieties. This value is directed by a number of factors including market opportunities, environmental constraints, consumption preferences, socio-cultural background, government policies (Keleman et al. 2013; Lacy et al. 2006; Montes-Hernandez et al. 2005; Teshome et al. 2007). The presence of these resources in the production systems might be deemed the reflection of the value that farmers assign to them. However, while targeting profitability of crop production which greatly depends on yield, farmers may overlook low yielding varieties and not show interest to the conservation of their genetic resources that might be useful today and tomorrow as potential resources for sustaining smallholders’ livelihoods or breeding programs. When facing drawbacks such as low inputs, low yield, market uncertainty, environmental heterogeneity, and risk factors (Brush 2000) farmers may not keep modern or local varieties in the production systems if they do not satisfy their needs and wants. This often happens when yield and profit are not achieved, and such varieties go off the system together with their associated local knowledge. If factors triggering the maintenance of these resources in the production systems are not well understood, it may be difficult to apprehend why, how and when these resources are lost. Farmer’s knowledge and perception of genetic resources are central to the *conservation through use* approach whereby the availability of these resources is ensured and increased (Neto et al. 2013). Such knowledge if well understood offers a valid window towards sustainable implementation of conservation and utilization strategies.

In pineapple [*Ananas comosus* (L.) Merr.] production systems of southern Benin the expansion of areas under cultivation exhibits a situation in which the crop genetic diversity is shrinking (authors personal observations after a country-wide pineapple collecting activities). Pineapple is the second tropical fruit in the global trade and contributes to over 20% of the world production of tropical fruits with 17 million tons (FAO 2012). In West Africa, Benin is the second major pineapple producer with 160,000 tons in 2011 after Nigeria (FAO 2013). An estimate of profit per hectare showed that pineapple crop provides higher margin to farmers than food crops (Tidjani Serpos 2004). However, the quantity traded globally is a small fraction of domestic production. Although the volume of pineapple produced increases over the years, the proportion of fresh pineapple exported to international market appears being still less than 2% (Arinloye et al. 2012; Fassinou Hotegni et al. 2012). One of the reasons explaining this situation was related to the heterogeneity in fruit quality and poor compliance with quality standards due to inadequate cultivation practices (Fassinou Hotegni et al. 2012). This situation leads farmers to either deliver their pineapple to the local market (Arinloye et al. 2012) or stop growing some “non promising” varieties. Meanwhile, conservation and use of genetic resources have barely been documented.

With very dynamic production systems (Adossou 2012) combined with the arising issue of pests and diseases (Fanou and Adekan 2006), the use of genetic resources and the decision to grow a specific cultivar depend on how much knowledge farmers have and how they link their specific constraints to the use of appropriate planting material. In other words farmer’s choice of variety might be guided by drivers that need to be scrutinized to recommend adequate conservation strategy. For instance, it is a common knowledge that in the pineapple cultivation system of southern Benin (the main production area) local old cultivars gave way to recently introduced cultivars such as Smooth Cayenne and Sugarloaf although scientific evidences are yet to be provided.

Pineapple farmer’s knowledge and rational behind the use of genetic resources are rarely assessed. Moreover, the complexity of such knowledge particularly when this is related to the biological material and the production systems calls for thoughtful approach whereby ethnographic studies can help understand farmer’s knowledge, priorities and needs in the choice of genetic resources (Temudo 2011). In these dynamic production systems, conservation strategies should be developed to maintain crop diversity. Specifically, answers should be provided to questions such as: what drives farmer choices of pineapple varieties and how this helps conserve genetic resources? Is the choice of variety guided by cultivation practices and how this is affected by farmer’s plant knowledge?

In this study we assume that farmer’s variety choice is guided by criteria such as cultivar traits, cultivar commercial value, and consumer preference while farmer’s knowledge of cultivation practices is intrinsically related to factors such as age, education level, location of the pineapple farm, and farmer’s knowledge of the plant. Our objectives were to (1) clarify the ongoing trends in varietal choice in the pineapple production systems and (2) understand factors affecting such choice so as to identify adequate conservation strategy to halt the risk of reduction of crop genetic diversity in the pineapple production systems in Benin.

## Materials and methods

### Study area

**Figure 1:**
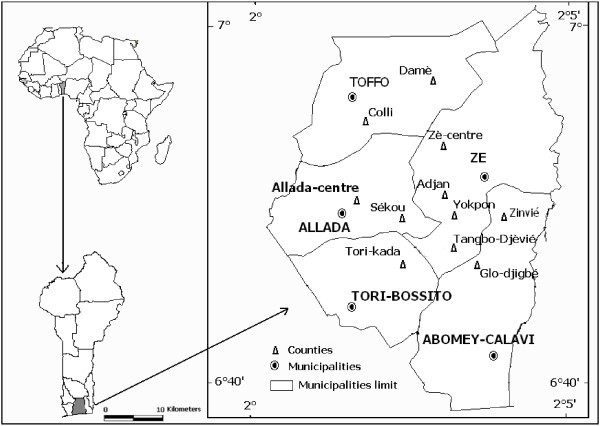
**Pineapple farming sites surveyed in southern Benin.**

The study was carried out from September 2012 to June 2013 in thirty four villages of eleven counties located in five municipalities of Southern Benin namely Allada, Tori, Toffo, Zê and Abomey-Calavi known as the pineapple production areas in the country (Figure 1 and Table 1). South Benin is located in the Guinean phytogeographical region (White 1986) with a semi-deciduous rainforest zone on ferralitic and lateritic soils, vertisols and hydromorphic soils (Azontondé 1991). It covers 17,920 km^2^ (Arouna et al. 2011) extending from the Atlantic coast and stretching between 1°45’ and 2°24’E and 6°15’ and 7°00’N to the west and 6°15’ and 7°30’N to the east. The area is characterized by a sub-equatorial climate with two rainy seasons and two dry seasons. The mean annual rainfall varies from 950 to 1400 mm covering 240 days. The mean annual temperature is 26°C (±2.5°C). The local economy is based on agriculture with production systems dominated by maize, cassava, oil palm and pineapple.

**Table 1:** **Cultivated areas and number of pineapple producers per municipality in the Atlantic department (MAEP** 2012 **)**

Municipality	Cultivated areas (ha)	Number of pineapple farmers
Abomey-Calavi	934	837
Allada	1569	1551
Ouidah	21	77
Toffo	5086	505
Tori Bossito	295	169
Zê	5981	1195

### Farmer sampling and data collection

In total, 177 producers were selected using a snowball approach. We included both members of farmers’ association and non-members; and also newly engaged and individuals with long experience of pineapple farming. Respondents underwent surveys in two phases with two questionnaires. The first questionnaire were related to socio-demographic data, production systems (e.g. land tenure, varieties, fertilization, pests and diseases management, market and income, constraints and opportunities); and the second questionnaire used ethnobotanical approaches to assess producers’ perceptions in terms of variety preferences, botanical traits, and agronomic practices. The second questionnaire was organized in six constructs, each consisting of 3 to 9 questions (items) related to knowledge on leaves, flowers, fruits, fertilization, irrigation and growing seasons identified through literature search and the first survey phase. Questions in the constructs were reflected as statements, and producers were asked to indicate their level of agreement using a 5-point Likert response scale, ranging from strongly disagree to strongly agree (Fanou-Fogny et al. 2011). The questionnaire was pretested with 10 farmers (who did not participate in the research) to ensure that the questions were understandable. Before each interview, we clarified the response scale using an example to ensure that participants understood the Likert scale. The constructs help assess farmer’s knowledge of pineapple using descriptors of leaves, flowers and fruits; and farmer’s knowledge of cultivation practices. Farmer’s knowledge of plant was deducted as a sum of scores resulting from farmer’s knowledge of leaf, flower and fruit traits while farmer’s knowledge of practice was obtained by adding the scores resulting from knowledge of practices such as fertilization, irrigation, and seasonality. Preference data were collected using direct scoring matrix with criteria such as fruit size (size), fruit form (ffor), fruit shelf life (cons), fruit commercial value (comv), flesh colour (clri), skin colour at maturity (clrm), fruit aroma (arom), consumer’s appreciation (capr), fruit taste (tast), number of propagules (nbrj). Propagules here include suckers and hapas. Preference scores vary from 10 to 1 with 10 being the highest mark.

### Data analysis

Descriptive statistics were used to examine farmers’ sociodemographic characteristics and to compute the median score of the constructs. Multiple item constructs were tested for the reliability of the questions and internal consistency using Cronbach’ α and item-total correlation. The items within a construct were regarded as consistent when Cronbrach’ α and the item-total correlations were higher than 0.80 and 0.30 respectively. Spearman correlation was used to assess the bivariate association between farmer’s knowledge of plant and knowledge of cultivation practices. Multiple linear regressions were performed to determine the contribution of the social attributes (e.g. farmer’s age, experience in pineapple production, locality, and education level) or farmer’s plant knowledge (independent variables) to farmer’s knowledge of cultivation practices (dependent variable). Principal Components Analysis (PCA) was used to group farmers with regards to preference criteria. This was done using preference scores. Data were analysed using R version 2.15.2 (R Developement Core Team 2013).

## Results

### Pineapple production system

**Figure 2:**
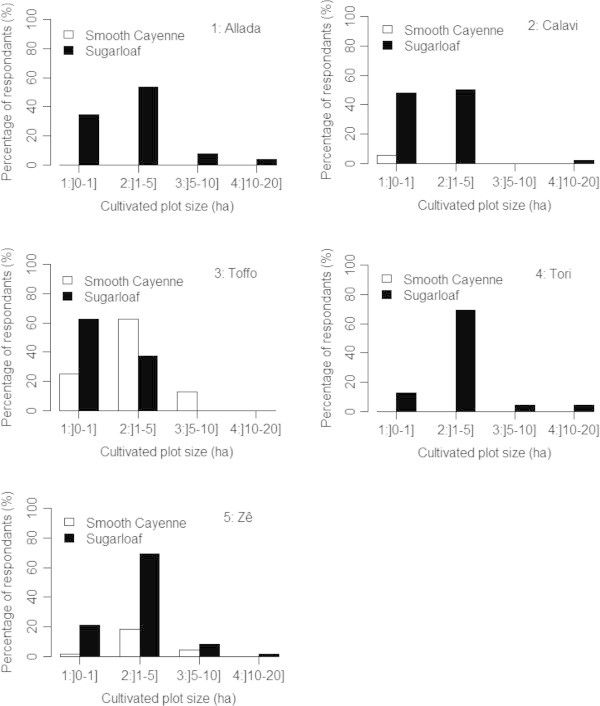
**Distribution of farmers according to land allocation (ha) expressed as classes per cultivar and production site.**

Pineapple farmers were in average 33.8 ± 8.3 years old. About 60% of them aged between 20 and 35 years old, 35% between 36 and 50 while less than 4% aged above 50 (Table 2). On average farmers were involved in pineapple production for the last ten years with 2 years for the least experienced farmer and 20 years for the most experienced one. About 50% of them are illiterate, 23% attended primary school and 18% reached secondary school (Table 2). Most farmers were male (96%). Only six female farmers representing 4% of respondents were surveyed as a reflection of the fact that men dominate the pineapple production system in Benin. Land tenure presented two major features; land was either owned through purchase or heritage (70% of farmers) or rented (97%). Farmers who owned land also rented additional plot for pineapple cultivation. Two pineapple varieties were produced in the study areas, namely Smooth cayenne and Sugarloaf. Local cultivars were rare and cultivated on a very small scale and consequently not visible in the cropping systems although present. In majority farmers only cultivated Sugarloaf (79% of respondents). Those who produced Smooth cayenne only represented 2% while 18% of respondents produced both varieties (Table 2). Moreover, production of Smooth cayenne was restricted to two sites, Toffo and Zê where more farmers allocated substantial land for the production of this variety (Figure 2). Smooth Cayenne was well produced in Toffo, with 60% of respondents allocating between 1 and 5 ha to that cultivar (Figure 2). In Allada and Tori the production was dominated by Sugarloaf. In these two localities more than 50% of respondents allocated between 1 and 5 ha to sugarloaf production. The situation was almost the same in Abomey-Calavi although farmers (less than 5%) produced Smooth Cayenne on a very small scale.

**Table 2:** **Sociodemographic characteristics of pineapple farmers in southern Benin**

Sociodemographic characteristics	Frequency	Percentage (%)
Age		
20-35 years	107	60.45
36-50 years	63	35.59
>50 years	7	3.95
Education level		
Illiterate	102	57.62
Primary	42	23.72
Secondary	32	18.64
Gender		
Male	171	96.61
Female	6	3.39
Variety cultivated		
Sugarloaf only	140	79.09
Smooth cayenne only	4	2.25
Sugarloaf and Smooth cayenne	33	18.64

**Figure 3:**
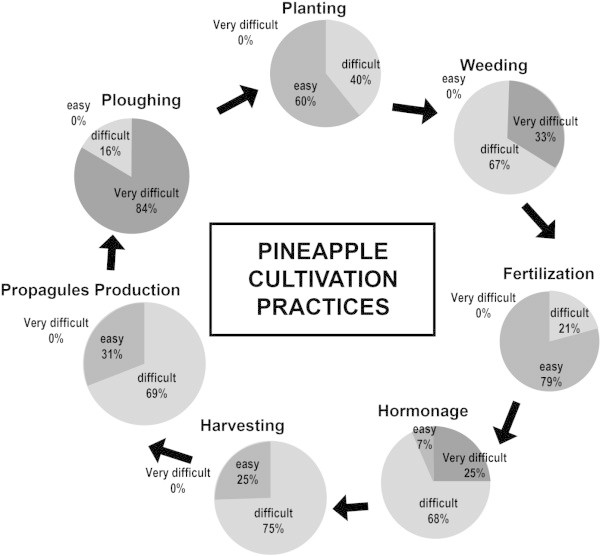
**Farmers’ cultivation itinerary in the pineapple production systems of Southern Benin.** Percentages indicate the rate of farmer’s expression of perception.

The main pineapple cultivation itinerary comprised land ploughing, planting, weeding, fertilization, forcing (hormone application), harvesting, and production of planting material. Cultivation practices varied from one cultivar to the other as described by Fassinou Hotegni et al. (2012). Most of these practices were labour intensive and perceived as difficult by farmers (Figure 3).

At harvest, it was noticed that 84% of the farmers sold their pineapple to the local market. About 50% of them targeted regional market while only 15% were involved in international trade. According to farmers, Smooth cayenne is international market-oriented while Sugarloaf mainly targets domestic and regional markets. All farmers recognized that Smooth cayenne generated more income (USD 5,750/ha) than sugarloaf (USD 3,950/ha) in the production systems of southern Benin. However, there are some bottlenecks to compliance with quality standards and market growth. These include heterogeneity in fruit shape and weight due to inappropriate cultivation practices (recognized by 80% of farmers) and the lack of adequate planting material (100% of respondents).

### Farmer’s knowledge of plant and production practices

Cronbach’ α coefficient ranged from 0.65 to 0.83 showing medium to high reliability of the questions (items) of the constructs (Table 3). The median scores for all constructs ranged from 14 to 35. The high value of median scores in comparison with the range of possible score showed that most farmers agreed on the items and shared relatively similar knowledge (Table 3).

**Table 3:** **Internal consistency and median scores of the six constructs related to farmer’s plant and cultivation technique knowledge**

Constructs	Number of questions	Cronbach α	Median	25th-75th	Range
Farmer’s leaf knowledge	4	0.72	19	18-20	7-20
Farmer’s flower knowledge	7	0.83	35	32-35	21-35
Farmer’s fruit knowledge	9	0.80	44	42-45	32-45
Farmer’s fertilization knowledge	5	0.80	21	18-23	10-25
Farmer’s season knowledge	5	0.65	22	20-23	14-25
Farmer’s climate knowledge	3	0.76	14	13-20	9-15

Spearman correlation test on socio-demographic attributes showed positive correlations between farmer’s knowledge of pineapple leaves, flowers, and fruits; and farmer’s knowledge of fertilization, season, and climate (Table 4). It was also noticed a positive relationship between farmer’s plant knowledge and cultivation practices. Moreover, there were significant correlations between farmer’s knowledge of fertilization and farmer’s knowledge of plant on the one hand and farmer’s knowledge of fruit and farmer’s cultivation practices on the other hand.

**Table 4:** **Correlation matrix of farmer’s plant knowledge and pineapple cultivation knowledge: leknow: leaves knowledge; flowknowl: flowers knowledge; frknow: fruit knowledge; feknow: fertilization knowledge; irknow: irrigation knowledge; clknow: climat knowledge; plknow: plant knowledge; prknow: practice knowledge**

	flknow	frknow	feknow	irknow	clknow	plknow	prknow
leknow	-0.075	-0.175*	-0.078	-0.009	0.078	0.433**	-0.014
flknow		0.046	0.037	0.158*	-0.162*	0.452**	0.044
frknow			0.750**	0.060	-0.070	0.687**	0.580**
feknow				-0.051	0.012	0.548**	0.750**
irknow					0.169*	0.114	0.507**
clknow						-0.048	0.467**
plknow							0.459**

*significant, **highly significant.

The relative contribution of drivers to farmer’s cultivation knowledge is shown in Table 5 which indicates three models. Model 1 and 2 revealed the contribution of socio-demographic features to farmer’s plant and cultivation knowledge while model 3 estimated the contribution of farmer’s knowledge of plant to farmer’s knowledge of cultivation practices. In model 1, locality of origin explained a big part of the variability observed in cultivation knowledge (standardized β = 0.64, p < 0.05). In this model the standardized β of variables such as experience and education level are close to significant (p = 0.06 and p = 0.05 respectively). Likewise in model 2, 27% of the variance in farmer’s plant knowledge could be explained by the locality of origin (standardized β = 0521, p < 0.05). In Model 3 farmer’s plant knowledge accounted for 33% of the variance in farmer’s practice knowledge, but only farmer’s knowledge of fruit showed significant β (standardized β = 0.596, p < 0.05).

**Table 5:** **Predictors of farmer’s knowledge in the pineapple production systems of South Benin**

Socio demographic characteristics	Standardized β	P	R^2^	Adjusted R^2^
*Model 1*				
Dependent variable: practice knowledge			0.47	0.45
Age	-0.73	0.29		
Experience	0.12•	0.06		
Locality	0.64*	0.99		
Education level	0.37•	0.05		
*Model 2*				
Dependent variable: plant knowledge			0.29	0.27
Age	0.10	0.90		
Experience	0.21	0.79		
Locality	0.52*	0.00		
Education level	0.12	0.10		
*Model 3*				
Dependent variable: practice knowledge			0.34	0.33
Farmer’s leaf knowledge	0.09	0.14		
Farmer’s flower knowledge	0.24	0.70		
Farmer’s fruit knowledge	0.59**	0.00		

• close to significant, *significant, **highly significant.

### Variety preference and knowledge of pineapple genetic resources

**Figure 4:**
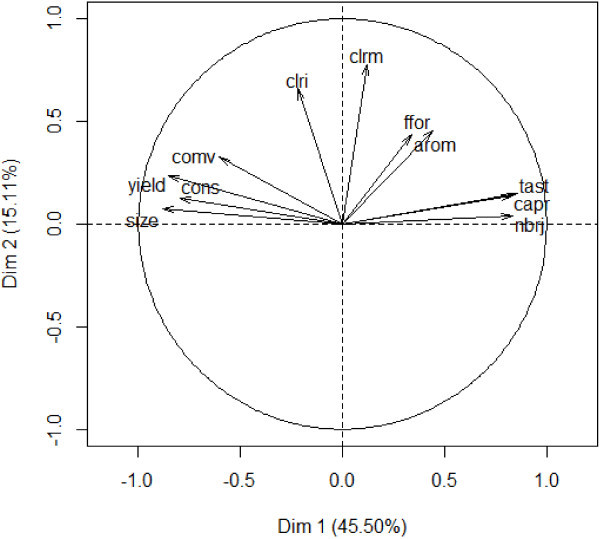
**Correlation circle of the pineapple characteristics with the agronomic and commercial criteria.** Fruit size (size), fruit shape (ffor), fruit weight (yield), fruit shelf life (cons), fruit commercial value (comv), flesh colour (clri), mature skin colour (clrm), fruit aroma (arom), consumer’s appreciation (capr), fruit taste (tast), number of propagules (nbrj).

**Figure 5:**
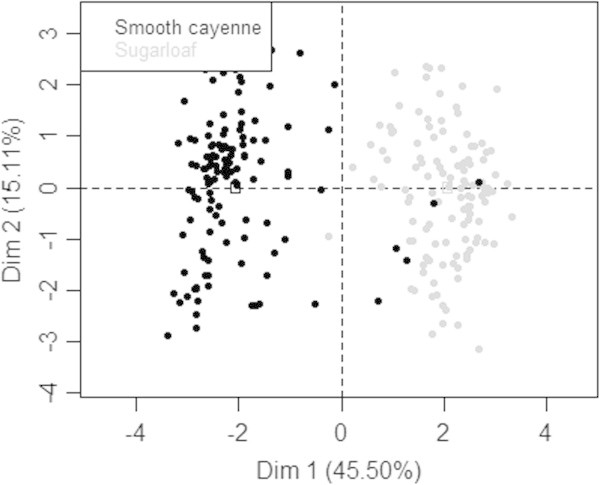
**Projection of the producers in the correlation circle.** Smooth Cayenne producers are in black colour and Sugarloaf producers are in gray.

Analysis of relationships between preference criteria indicated high and positive or negative correlations between criteria (Table 6). For instance, high significant correlations were observed between variables such as consumer’s preference (capr) and taste (tast), consumer’s preference and number of propagules produced (nbrj) while significant negative correlation was noticed between fruit weight (yield) and number of propagules. Principal Components Analysis revealed two major components that explained together 60.61% of the total variation of pineapple agromorphological traits (Figure 4). The first axis (explaining 45.5% of total variation) was positively correlated with criteria such as the number of propagules (nbrj), the fruit taste (tast) and the consumer’s preference, and negatively correlated with other criteria such as fruit size (size), fruit weight (yield), fruit shelf life (cons), and fruit commercial value (comv). The second axis (with 15.1% variation explained) was rather positively correlated with the fruit flesh colour (clri) and the fruit skin colour at maturity (clrm).The representation of respondents in the factors map showed two groups of farmers (Figure 5). The first group included farmers who preferred pineapple cultivars that bear big fruits, with high commercial value, and high shelf life. These criteria were attributed to Smooth cayenne. The second group comprises farmers who preferred pineapple with high consumer preference value, good taste and high number of propagules. These criteria were attributed to Sugarloaf. All surveyed farmers can easily identify Smooth cayenne and Sugarloaf. According to them leaf traits (e.g. presence of spines, length, and width) and fruit traits (length, size, form and colour) are used to distinguish Smooth Cayenne and Sugarloaf.

**Table 6:** **Correlation matrix of preference criteria**

	arom	clri	capr	size	nbrj	yield	clrm	ffor	cons	comv
tast	0.30**	-0.09	0.75**	-0.71**	0.69**	-0.67**	0.13	0.42**	-0.58**	-0.37**
arom		0.05	0.30**	-0.25**	0.18**	-0.17*	0.21**	0.23**	-0.16*	-0.10
clri			-0.05	0.20**	-0.08	0.37**	0.32**	0.01	0.10	0.12**
capr				-0.63**	0.68**	-0.62**	0.15*	0.32**	0.58**	-0.43**
size					-0.70**	0.80**	-0.08	-0.30**	0.69**	0.47**
nbrj						-0.62**	0.04	0.29**	0.66**	-0.42**
yield							0.37**	-0.32**	-0.62**	0.51**
clrm								0.30**	0.00	0.06
ffor									0.23**	0.06
cons										0.54**

*significant, **highly significant.

Fruit size (size), fruit form (ffor), fruit weight (yield), fruit shelf life (cons), fruit commercial value (comv), flesh colour (clri), mature skin colour (clrm), fruit aroma (arom), consumer’s appreciation (capr), fruit taste (tast), number of propagules (nbrj).

## Discussion

### Production systems and polarization of farmer’s knowledge and preferences

Our data showed that the pineapple production was dominated by men. There are two reasons that may explain this situation. First, as argued by Royer and Bijman (2012) and according to farmer’s perception, pineapple production is labour intensive and requires a lot of manpower. Second, women are usually restricted from land heritage in many parts of Africa and particularly in Benin (Achigan Dako et al. 2008; Lastarria-Cornhiel 1997). Land tenure system is similar to what is observed elsewhere in the country whereby farmers acquire land through heritage, purchase, and renting. In the pineapple production system, land acquisition is mainly by renting (90% of respondents) even if 70% of the surveyed farmers were owners. The renting of additional land may mean that farmers are seeking higher production. Alternatively, this situation suggests that most farmers are resource limited and cannot acquire their own land for production. It is also probable that some of them are temporarily growing pineapple and might quit at any time when constraints become severe. In fact, the system is dominated by young farmers (30 years old in average) in search for opportunities and might not necessarily invest for the long term. Moreover, the study revealed that the majority of the producers allocated 1 to 5 ha to pineapple production. This points out the issue of economies of scale where farmers individually cover small size of land scattered over different villages, as response to constraints currently faced for land access in southern Benin (Mongbo and Floquet 2006).

Although a high level of illiteracy was recorded among farmers, indicating a low-educated community, farmer’s knowledge of cultivation practices and plant traits was consistent as revealed by Cronbach’ test. This indicates that in general farmers share similar knowledge. However, our study did not specifically assess the content of the knowledge. Finding effective knowledge is not similar to finding commonly held knowledge (Bart 2010). Effective knowledge might be limited to a few farmers who are members of a pineapple growers association and have in general adequate information. They are aware of opportunities and participate in training organized by other institutions. These farmers know the cultivation techniques required to improve the quality of their products. However, some of them may not implement the knowledge acquired because of lack of financial support (Royer and Bijman 2012).

Farmers choose which crop varieties to grow taking into account a range of biophysical, social and economical environment over space and time (Lacy et al. 2006). Improved understanding of farmer’s varietal choices is paramount to fruitful collaboration between farmers and scientists (Lacy et al. 2006). In Benin, the pineapple cropping system is split in two, based on the varieties grown (Fassinou Hotegni et al. 2012). These varieties (Smooth Cayenne and Sugarloaf) are modern introduction and are well differentiated from local old cultivars that are lesser and lesser visible in the production system. Both varieties present distinctive physical and agronomical traits (Fassinou Hotegni et al. 2012). An incomplete understanding of why farmers choose one variety or the other will continuously generate considerable difficulties when developing viable options to reduce fruits heterogeneity. Our results showed that Sugarloaf was the most cultivated variety (on 405 ha against 90.25 ha for Smooth cayenne according to respondents data only), which was produced by about 80% of farmers. Only 2% of farmers exclusively produced Smooth cayenne and 18% diversify their pineapple production systems by cropping both varieties. This trend also was observed by Arinloye et al. (2012) who related the low Smooth Cayenne production to the low fresh pineapple export by Benin. Moreover, the production of Smooth cayenne has shrunk to two localities (Toffo and Zê) where farmers still have adequate knowledge of cultivation techniques to participate in international market. Farmers of these localities allocate more land to the production of this variety compared to Sugarloaf as found by Arinloye et al. (2012). Here, farmers do not necessarily grow the variety that gives the highest profits per unit area as stipulated by the neoclassical economic model (Small 2002), they take into account a number of other criteria that guide their decision even if they grow just one variety.

Results revealed that farmers split in two groups based on variety preference criteria such as fruit size and weight, shelf life, fruit commercial value, consumer preferences, and number of planting material (slips, hapas, suckers) produced. A first group of farmers prefers Smooth cayenne because it produces bigger fruit with high price on the international market and with high shelf life. The second group of farmers prefers Sugarloaf as this variety is sweeter and well appreciated by local consumers. We can speculate that farmer’s varietal choice is certainly a measure of insurance against the stringent international market standard albeit this later is more profitable.

Farmer’s varietal choice explains the zonal polarization of the cropping system and influence farmer’s knowledge of cultivation techniques. This knowledge depends on the location of the pineapple farm as revealed by the multiple regression analysis. Incidentally, the localities with more varieties were those with higher production. In these sites, 60% of farmers have more than 15 years experience in pineapple production. They also hosted educational programs in pineapple production. This indicates the role of the farmer’s knowledge in agricultural development (Yassin et al. 2002) and justify that high agricultural production is often linked to farmers’ access and use of agricultural knowledge (Briggs 2005; Feder and Savastano 2006).

Farmer’s knowledge of cultivation techniques also correlated with their knowledge of the plant (specifically knowledge of fruit) which confirms the idea that cultivation practices are variety dependent (Fassinou Hotegni et al. 2012). Many farmers mostly grow Sugarloaf because it can easily be sold in local and regional market and requests low investment and without quality control requirement (Arinloye et al. 2012). High yield and high commercial value do not always guide the choice of variety. Other criteria such as consumer preference, market accessibility, handling of cultivation practices guide the choice of farmers. In addition, farmers rely on market diversification as a protective strategy to safeguard their investments (Arinloye et al. 2012; Wilson 1986). Although farmers are aware of the constraints facing them, they cannot do much to change the situation. This shows the need for public sector to support capacity building and provide other incentives (e.g. facilitating the setting up of processing factories, facilitating access to financing) to pineapple farmers.

### Implications for conservation and utilization of pineapple genetic resources

Farmers are the primary creators, users and conservers of crop genetic resources on farm. Their decision making processes influence the level, status and dynamics of inter and intra specific diversity and management practices (Teshome et al. 2007). If international market remains stringent (the contrary is not expected) smallholder farmers will continue shifting from labour intensive varieties (Smooth cayenne) to more easy-to-grow varieties (Sugarloaf). This trend will persist not only because farmers are guided by local consumer’s preference but more importantly because farmer’s knowledge of cultivation techniques is intrinsically related to their knowledge of the plant. Consequently, as cropping systems are locality-dependent and cultivation practices variety-dependent, the level of pineapple intra-specific diversity might be reduced in the absence of adequate measures. Our study clearly flagged out the factors that determine the place of pineapple varieties in the production systems and the interest that farmers have in them. These factors are important to develop a sustainable utilisation strategy (Brush and Meng 1998). In the pineapple production system in southern Benin the *conservation through use* approach needs to be sustained with a number of strategic actions such as 1) collection of pineapple genetic resources, 2) morphological and genetic characterization of these resources and definition of a core collection, 3) promotion of pineapple diversity with emphasis on production locations where this diversity is high, 4) training and capacity building, particularly on cultivation practices.

Establishment of a core collection of pineapple genetic resources including landraces is of paramount importance. Although pineapple was listed in 2007 by the government of Benin as a priority commodity in the country, no collection of genetic resources has been undertaken to secure germplasms. Moreover, there is no single genebank available for breeding programmes. Although pineapple is vegetatively propagated, we cannot only rely on farmers to maintain all the diversity that might be available particularly when this diversity is unknown. That is why in addition to germplasm collection activity the morphological and genetic characterization of pineapple cultivars should be carried out. Conservation of genetic resources both *in situ* and *ex situ* needs to be guided by information on the novelty of specific populations at the whole-genome and specific allele levels. To the best of our knowledge the genetic diversity in pineapple cultivated in West Africa has never been evaluated. It is possible that in addition to modern cultivars, such as Smooth cayenne and Sugarloaf, and traditional landraces other introductions might have happened the same way knowledge was imported from neighbouring countries (Royer and Bijman 2012).

Another strategic action includes promotion of pineapple diversity with emphasis on production areas where this is high. In developing countries, records reveal that research centres are not the best keepers of genetic resources particularly when it comes to vegetative crops which need to be maintained as live collection. With regards to this situation, pineapple genetic diversity should be promoted at community level. This could be supplemented with training and capacity building particularly on cultivation techniques. A challenge for agriculture and rural development agents will be to develop training and radio programs to help expand pineapple farmers’ knowledge and practical skills in order to improve pineapple production in Benin.

## Authors’ contribution

EGAD initiated the research protocol, supervised data collection, contributed to write the manuscript; CAA finalized the research protocol, tested the questionnaires, collected the primary data and conducted the survey, contributed to data analysis, initiated the first draft; SN contributed to protocol development and manuscript writing, NVFH, CA, and AA contributed to manuscript proofreading and improvement. All authors read and approved the final manuscript.
